# Addressing racial disparities in perinatal care for African American/Black individuals in the Chicago community health setting: a qualitative study

**DOI:** 10.1186/s12884-022-05100-4

**Published:** 2022-10-13

**Authors:** Jonathan Alhalel, Lane Patterson, Nicolás O. Francone, Sankirtana Danner, Cassandra Osei, Catherine Ann O’Brian, Laura S. Tom, Lisa Masinter, Elizabeth Adetoro, Danielle Lazar, Abbey Ekong, Melissa A. Simon

**Affiliations:** 1 Feinberg School of Medicine CHET (Center for Health Equity Transformation), Northwestern University, Evanston, USA; 2 Alliance Chicago Network, Chicago, USA; 3 Access Community Health Network, Chicago, USA

**Keywords:** Black/African American health, Women’s health, Health equity, Health disparities, Perinatal care, Healthcare, Qualitative research

## Abstract

**Background:**

There are persistent disparities in maternal and infant perinatal outcomes experienced by Black birthing persons compared with non-Hispanic white (NHW) individuals in the US. The differences in outcomes arise from not only socioeconomic factors and individual health behaviors but also structural racism. Recent research is beginning to elucidate the benefits of patient navigation to support underserved minoritized individuals who experience this constellation of barriers to equitable care. Qualitative research that utilizes both the experiences of Black birthing individuals and the expert opinion of healthcare providers working with them can serve to guide a patient navigation intervention to further decrease disparities in perinatal outcomes.

**Methods:**

We conducted 30 interviews between August and December 2020 with Black birthing individuals in the Chicago metropolitan area and healthcare providers who care for this population both in Chicago and across the nation to explore their experiences, perceptions of barriers to care and ways to decrease inequities.

**Results:**

Clinical care team members acknowledged the presence of health disparities experienced by Black pregnant individuals compared with their NHW counterparts stemming from racism, discrimination, and lack of resources. Patients similarly reported personal experiences with these disparities and barriers to care. The successful methods used by clinical care teams to help decrease these differences in the past included patient education on important topics such as breastfeeding and the use of patient advocates. Effectively screening for social determinants of health by someone the patient trusts was also cited as important. Regarding perinatal care practices, clinical care team members described the importance of patient education needs and care team cultural competency. Patients’ reported positive and negative experiences corroborated these findings, emphasizing the importance of trust, listening, education, access to care, support, and patient advocacy. Finally, the care team members and patients agreed that active trust-building can help the provider/patient relationship and ultimately improve outcomes.

**Conclusions:**

These qualitative research findings improve the understanding of barriers to care and will help guide development of an intervention to reduce the health disparities experienced by Black pregnant persons.

## Background

Significant and egregious disparities have persisted in maternal and infant perinatal outcomes experienced by Black/African American (hereafter referred to as Black) birthing persons compared with non-Hispanic white (NHW) individuals in the US for decades [1]. Data show that pregnancy-related maternal mortality rates have been worsening in the US, with the rates more than doubling over the past decade [2]. Mortality rates in Black persons account for one-third of all pregnancy-related deaths in the US [3] and are more than three times higher than those of NHW persons [2]. Pregnancy-related complications are closely tied to infant morbidity and mortality. Preterm birth is a significant cause of racial disparities in infant mortality [4], and preterm birth rates are 50% more common in Black persons compared with NHW persons in the US [5] and have risen by 3% in Black persons since 2014 [6]. Preterm birth as well as other factors contribute to a 2.5 times greater infant mortality rate in Black infants compared with NHW infants [7].

Disparities in maternal and infant morbidity and mortality experienced by Black persons in the US are due to multiple factors. For purposes of this report, maternal health disparities are defined herein as the overall differences observed in health outcomes inclusive of inequities (unjust and preventable differences). perinatal care allows persons to receive attention to conditions than can lead to high maternal morbidity and mortality and access to support and referrals to resources for addressing Social Determinants of Health (SDH) [8, 9]. The CDC defines SDH as “conditions in the places where people live, learn, work, and play that affect a wide range of health risks and outcomes.”[10] As recognized by the American Public Health Association (APHA), “Racism is a driving force of the social determinants of health and a barrier to health equity [11]. Black individuals are less likely to receive care in the 1^st^trimester [12], and the majority of high-risk patients receive < 80% of the components recommended for their needs including depression screening, referral to case management, and referral to the Women, Infants, and Children (WIC) program – a federal assistance program that safeguards the health of low-income pregnant individuals, breastfeeding individuals, and children under the age of 5 by providing nutritional information, supplemental foods, and healthcare referrals [13].

Barriers to receiving adequate perinatal care are especially pronounced for Black individuals and stem from multiple levels of influence (e.g., individual, interpersonal, structural). These barriers include systems barriers, structural barriers, psychosocial barriers, and low health literacy, among others [14–16]. Notably, socioeconomic factors and individual health behaviors are insufficient to explain these disparities [17]. Structural racism, such as in the form of residential redlining [18] and cumulative stress from racism [19], is thought to be a significant contributing factor to maternal morbidity and mortality in Black persons [18, 19]. Current perinatal care practices may need to be redesigned to address SDH and barriers at multiple levels of influence in order to address persistent and widening health disparities experienced by Black birthing persons.

In an effort to address SDH as contributing factors in perinatal health outcomes of Black persons, national and local organizations are attempting to redesign perinatal care for these individuals. The American College of Obstetricians and Gynecologists (ACOG) Task Force proposed a “fourth trimester” and recommended tailoring of postpartum care to a birthing person’s individual needs [20]. Family-centered care, including provision of a doula, midwife, and lactation specialist to minoritized birthing persons by minoritized individuals through a non-profit organization, has supported birthing persons to have healthy pregnancies and deliveries [21]. Patient Navigation (PN)—an empirically-supported strategy initially deployed to address racial/ethnic cancer health disparities [22, 23]—has demonstrated efficacy in overcoming barriers to healthcare access and utilization, strengthening patient linkage to local/community resources, improving consistency of care, and addressing SDH among Black birthing persons and other underserved minoritized individuals across a range of health-related contexts [24]. Research is emerging that demonstrates the potential of patient navigation in supporting underserved minoritized individuals in their perinatal care. For example, one patient navigation study conducted at a low-income ambulatory care clinic demonstrated significant improvements in postpartum visit adherence and depression screening, vaccination of influenza and HPV, and contraception uptake among participants [25–27].

The need for redesigning perinatal care to address the disparities Black persons experience is undeniable, given how egregious and persistent these disparities are. To do so requires an in-depth understanding of the unique needs of the patient population as well as key stakeholders including physicians, nurses, social workers, and community health workers and advocates who will hereby be called clinical care teams. This study explores patient experiences with perinatal healthcare and providers’ thoughts on the causes of Black maternal health inequities, their particular role in providing care to Black persons, and methods to curbing maternal health disparities in the Black communities they treat. Findings from these qualitative interviews were used to inform the development of the OPTIMIZE intervention—an implementation trial at Federally Qualified Health Centers (FQHCs) which has the goal of reducing maternal health inequities in Black pregnant persons.

## Methods

### Study design

Qualitative methods, such as patient and clinical care team member interviews, are valuable for collecting meaning-centered, contextually based data. We conducted patient and clinical care team interviews, soliciting Black pregnant or postpartum patients’, obstetrics providers’ and healthcare leaders’ opinions and perspectives on barriers to perinatal care for Black individuals, including SDH, as part of formative work for a parent study – an intervention implementation trial of perinatal patient navigation among Black prenatal and postpartum persons in Chicago. Leveraging the Consolidated Framework for Implementation Research (CFIR), we designed the interview questions to elicit responses that would guide the implementation of the OPTIMIZE trial and refine the intervention [28]. CFIR proposes a constellation of constructs in five domains of program implementation factors, including 1) intervention characteristics, 2) inner setting, 3) outer setting, 4) characteristics of intervention implementers, and 5) implementation processes [29]. A diverse multidisciplinary study advisory committee, composed of staff from research teams, maternal and child health programs, EPIC teams, quality improvement staff, and others – some of whom are also minoritized birthing persons – guided the development of the interview guides. Weekly meetings were held to iteratively develop the interview guides with questions to help explore the current condition of Black persons’ perinatal healthcare experiences, the barriers present to their care, and approaches that can mediate these barriers. This committee reviewed each question from the iterative versions of the guide to ensure that it 1) aligned with constructs from CFIR, 2) used appropriate language that all respondents could comprehend, and 3) would elicit the key information needed to refine the intervention. Furthermore, to explore the clarity and language of the questions, practice interviews were conducted with members of the research team [30]. See Tables 1 and 2 for sample items from the qualitative instrument. All study procedures were approved by the Northwestern University Institutional Review Board (IRB).

**Table 1:** Clinical care team member interviews

Section	Sample Question
Perceptions of Perinatal Care for Black Birthing Persons	What do you think is the role of trust in the provision of effective perinatal care for African American and Black patients?
Quality Improvement	Have you been involved in previous change initiatives for African American and Black patients in perinatal care?
Services/Outside Supports	What kinds of services or programs exist at your organization during the pregnancy and the postpartum period?
Social Determinants of Health	Do you or others on your team regularly screen pregnant patients for social determinants of health needs? This may include things like financial difficulties, housing, food insecurity, childcare, safety/domestic violence/interpersonal violence, transportation, utilities, or education
Current Perinatal Practices	What are areas of perinatal care that you wish you could address but currently don’t address either because of time, lack of a workflow/system, capacity, or some other reason?

**Table 2:** Patient interviews*

Section	Sample Question
Perinatal Care, Postpartum Care, and Accessing Healthcare	Would you say that you trust/trusted your prenatal care clinician? What led you to (not) feel this way?
Postpartum Care	As much as you’re comfortable, how would you describe your postpartum experience? What type of materials or other information did you receive that was most helpful?
Beliefs about Health and Health Behaviors	What does a healthy pregnancy mean to you?
Prior Experiences with Receiving Support or Overcoming Barriers to Accessing Healthcare During and After Pregnancy	What support or resources have been/were provided to you about your concerns during your pregnancy?

### Sample interview questions

#### Study setting

The study was conducted virtually, due to the COVID-19 pandemic, with participants recruited from the Chicago area and nationally. Chicago participants include persons connected with AllianceChicago and ACCESS Community Health Network, two community health center networks that provide care to primarily underserved, racially and ethnically diverse populations. National partners included organizations that conduct work focused on Black maternal health.

### Recruitment

A purposive sample of participants was recruited via nomination by community partners and our study team. During this process, site leads from our participating community health centers distributed email invitations to a broad ranges of clinical care team members who work with pregnant patients to invite them to the study. Site leads similarly identified and reached out to patients receiving care at their respective community health center to invite them to participate.

Eligible participants for patient interviews included those who were: (a) 18 years of age or older (b) identified as Black or African-American and (c) pregnant or had recently given birth. As clinical care team interviews were conducted as formative work to inform design and implementation of a perinatal patient navigation program, eligible participants included those who were: (a) 18 years of age or older and (b) had professional expertise and/or in occupations involving the health with Black pregnant individuals. Hereafter referred to as “clinical care team members,” a diverse range of professionals was included, e.g., obstetrics providers, health center staff, and leaders from community-based organizations.

Four trained project staff members approached all clinical care team membersby email between August and December 2020 to schedule interviews. Due to the COVID-19 pandemic and the need to conduct interviews remotely, this study’s procedures were approved by the Northwestern University Institutional Review Board to be conducted via Zoom. Study staff were also approved to obtain verbal consent from participants. Interested participants were sent the copy of the informed consent form via email and scheduled for a Zoom interview conducted using the semi-structured interview guide after providing consent.

### Data collection

A team of four trained research staff members conducted individual interviews, using the semi-structured interview guide for clinical care team members, consisting of questions on providers’ perceptions of perinatal care for Black birthing persons, current practices in relation to services and SDH screening provided to patients, and current perinatal practices. The patient interview guide was also semi-structured, consisting of questions on patient perspectives on perinatal care, trust, challenges with perinatal care, and health beliefs. The interviews, lasting between 20–60 min, were audio recorded. Participants were sent a demographic questionnaire as follow up. Participants received a virtual $50 gift card upon completion of their interview.

### Data analysis

Clinical care team interview recordings were transcribed verbatim using an online transcription service and reviewed for errors by researchers. An initial codebook that included predefined codes and themes was developed based on the semi-structured interview guide. Members of the research team (JA, NF, SD, CO) then independently reviewed transcripts to identify any new codes to add to the pre-defined codes and themes from the interview guide. This approach allowed for a combination of deductive and inductive coding which is most commonly used in qualitative studies [31]. Coding schemes were then compared and discussed until a consensus was reached about the higher-level coding scheme [32].

A rapid analysis process – a useful method for projects that are less than one year old and require real-time modification of the process – was used for coding all clinical care team interviews. A two-part method, including summarizing each transcript and later identifying commonly occurring themes, was adopted that has shown similar results to in-depth analysis [33]. More specifically, researchers summarized then dual-analyzed two interviews for early analysis. An intensive, team-based analysis approach to coding followed. Each interview was summarized and coded by two different researchers. They each pulled data for each interview by following the audio and transcript and compiling a master list of clinical care team member comments under each code or theme. Once the analysis of every interview was completed, each of the final coding categories mentioned above was looked at by one researcher to identify common themes. As is standard practice in qualitative research, we used qualitative descriptions and exemplar quotes to convey the breadth and strength of agreement with a statement, rather than quantifying responses [34].

## Results

A total of 30 interviews were conducted. Table 3 depicts the characteristics of Clinical care team members. Table 4 describes their roles. Most clinical care team members were Black (57.9%), female (90.5%), and had a medical degree (42.9%). Table 5 depicts the characteristics of patients. Most patients were Black (80%), female (88.9%) and had completed high school or some college (88.9%).

**Table 3:** Clinical care team members demographics

**Gender**	**n (%)**
Male	0 (0)
Female	19 (90.5)
Non-binary/third gender	0 (0)
Prefer to self-describe	0 (0)
Prefer not to say	0 (0)
Unanswered	2 (9.5)
**Race/ethnicity**	**n (%)**
White	2 (9.5)
Hispanic or Latino	2 (9.5)
Black or African American	11 (52.4)
Native American or American Indian	0 (0)
Asian / Pacific Islander	3 (14.3)
Other	2 (9.5)
Unanswered	1 (4.8)

**Table 4:** Clinical care team member roles

**Role**	**n (%)**
Program/Community Organization Directors	3 (14.3)
MAs	1 (4.8)
MDs	9 (42.9)
Nurses	3 (14.3)
Social Workers/Case Managers/Care Coordinators	5 (23.8)

*MD* refers to Medical doctors, *MA* to Medical Assistants

**Table 5:** Patient demographics

**Gender**	**n (%)**
Male	0 (0)
Female	8 (88.9)
Non-binary/third gender	0 (0)
Prefer to self-describe	0 (0)
Prefer not to say	0 (0)
Unanswered	1 (11.1)
**Race/ethnicity**	**n (%)**
White	0 (0)
Hispanic or Latino	0 (0)
Black or African American	8 (80)
Native American or American Indian	1 (10)
Asian / Pacific Islander	0 (0)
Other	0 (0)
Unanswered	1 (10)

There were 9 patients total. One patient identified as both Black or African American and Native American or American Indian

The key qualitative findings are organized around the categories and themes that emerged from our data analysis process, including: 1) provider awareness of perinatal disparities, 2) trust, 3) change initiatives, 4) services and resources, 5) SDH, and 6) perinatal care practices. A summary of our findings, including themes, subthemes, and exemplar quotes can be found in Table 6. The findings across interviews elucidate current perinatal practices and their level of success as reported by providers and patients They tell a story of the need for attention to SDH, the requirement to remove barriers to accessing perinatal care, and the utmost importance of trust in a patient-provider relationship.

**Table 6:** Interview results by category, theme, and exemplar quote

Categories	Themes	Clinical Care Team Members Exemplar Quotes	Patient Exemplar Quotes
Perinatal Disparities	Access to Care	“where I feel I see a lot of disparity is not having access to insurance that really decreases the quality of care. There are a lot of restrictions in terms of where patients can go with specific types of insurance or if they don’t have insurance at all.”	“that’s where they offered it.” “my insurance had canceled because of my age and then she wanted me to pay out of pocket.” “…and then once you get to doctor's appointments, Oh my God, Scheduling. Because it's like… today is December 7th. Well, [they] have a doctor's appointment for February the second.” “I live out South. So I'm like an hour ride to the North side. So when you tell me, Oh, I can't be seen. And I'm going to go all the way that back south to make a new appointment to come all the way back up North. That's just a no, no. Yeah. Especially when I'm on public transportation.” “I would [be] sitting up in front for two whole hours… it feels like I have the carve out my whole entire day”
	Racism	“racism and discrimination in the healthcare system… Black women not being listened to in the context of pain or discomfort. When they make those expressions sometimes they’re ignored and other times they are told it’s not that big of an issue. That issue in particular is something that has been happening even in the early days of the study of obstetrics and gynecology—we can sort of track back these institutional practices of healthcare providers where Black women’s pain and discomfort is ignored. It’s unfortunate we continue to see those same patterns today and there have been clear examples where that has actually led to maternal death for Black women.”	“I suggest to have someone there that can help advocate for you just based off what African-American women have been going through in hospitals.” “I’ve met some [providers] that just … don't care. They don't listen to you. They just say, this is this and this is your only option.” “constantly telling everyone the doctors,[ everybody], that something’s not right and no one's listening to me.” “ to be ignore[d] for that long, … that's what really troubled me.” “A lot of the times with healthcare workers, you know, you just feel powerless sometimes because you know, they're the doctor and you're not” “… A lot of the times with healthcare workers, you know, you just feel powerless sometimes because you know, they're the doctor and you're not….”
	Resources	“Particularly for low-income Black women, having the resources and support to link with social services they need, whether it’s transportation, housing, nutrition support, and mental health challenges and how and when women can access those support services can be a cause for anxiety.”	“so I was laid off, my job due to the virus… I had to continue to pay every time I had a prenatal visit, I had to pay $40”
Trust	Importance of Trust	“trust is the most core fundamental value that’s missing.”	“If I didn't trust them, I would not go… if I can't trust you, I know you're going to kill me… it may seem funny, but realistically, yeah.” “And to know that like I was able to have someone that was able to inform me, educate me, or especially on things that I didn't know, and to answer my questions…. It was definitely uplifting.”
	Establishing Trust	“if we talked more about trust – do you trust and feel respected by your healthcare provider – having that uncomfortable conversation can help the psychological fear of [physicians not looking out for their best interests]”	“she made sure that she was present” “I've been going to them in the same doctor before I actually did get pregnant.”
Change initiatives	Patient Education	“…orient them and prepare them for [breastfeeding] because often people don't understand that, you know, might be really difficult. You might have a hard time, it might take a lot of work. You don't produce enough milk, you're going to feel guilty.”	“I feel like when a doctor can talk to me, explain things to me…, then yeah. I can trust you when you tell me … next time I see you [I] want to have this or that.”
	Patient Advocates	“A lot of these positive examples [initiatives] from my perspective have largely come from community based perinatal support organizations like midwifery care or community-based doulas… they are centering Black women and other women of color in healthcare practice. What I have seen that has been key in their work is that they center women of color and their experiences.”	“I feel very supported by my doula and how she's set up. Like she even came to my home to do her own prenatal visits with me. It was part of her package. So she does prenatal visits. She of course will support me during and after labor and postpartum. She's gonna still help me with breastfeeding “I think the best is to have a doula. Get a doula. They help, they help a lot. Cause there's a lot of stuff you're not going to understand.”
Services and Resources	Beneficial Services	“I’ve had patients that have done the moms group and absolutely loved it. They were really invested, motivated, they got a lot of information.”	“everything will be needed and it’s best to get it when it's, you know, provided for you.” “they also provided and offered a virtual, like pregnancy plan where like, I can take pregnancy classes with other people online and everything.” “[they] went above and beyond and faxed [the company] all my appointments to make it easier for me. To ensure that I had a ride there whenever my appointment was, and I didn't have to call them every time I needed a ride.”
	Addressing patient Needs via Navigation	“I can’t say that for any individual patient that I refer to either of these things that I’m able to see the outcome.”	
Social Determinants of Health	Effect on Care	“particularly for low-income Black women, having the resources and support to link with social services they need, whether it’s transportation, sometimes housing can be a big issue, nutrition support, these are all issues that are related to health.”	“I was the only aware that it was provided, like after baby was born just for the baby where they, you know, informed me that no, you know, you can get it now. It's also for you too. And once I started getting it, it was a big benefit because, it provided fruits, vegetables, stuff that, that I wanted at that time.”
	Best Suited to Screen	“I feel like whoever the patient trusts, like whoever [healthcare worker] feels like they’re building rapport with that patient and asking those questions are going to yield meaningful answers.”	“I was telling her my situation about me not working and everything with the job. So she recommended me to apply for County care, like a medical card… She gave me the information, I applied online and then I got the card within like two to three weeks.”
	Screening Frequency	“So if I’m implementing a screening questionnaire, that’s not a waste of my time because the information is important, but I don’t think it’s an optimal use of my time.”	
Perinatal Care Practices	Time Devoted to Patient Care	“I think we should extend our hours every day instead of just two days a week.”	“But most importantly, it was just, they gave you the things in the tools you needed to know,”
	Cultural Competency	“You have providers who are not accustomed to working with African American patients who don’t always understand dialects/lingo and don’t utilize language that patients can always understand, which can have a major impact on patient education and their understanding of what exactly their medical condition is.”	“So I know by now what should be going on with my body when I'm pregnant.” “brush up on the newest things that's going on in our, you know, in our world to, you know, be basically be aware of the new stuff and you know, each generation’s change.” “not … even when you think you’re a veteran mom, you think you know, but you don't cause everything changes over the years”
	Mental Health	“Some of the cultural stigma around mental health and strong black woman syndrome… can really be a barrier in terms of acknowledging that there are challenges [mental health] and that we should talk to someone.”	“prenatal care is seeing to it that I am all around taken care of physically and emotionally”
	Expectations for Labor and Delivery Education	“I think a lot of expectations in labor and postpartum we should address ahead of time, globally for patients, but especially for patients that might not have as much trust in the health system… maybe around 30, 32 weeks, labor wise, here’s all the things that might happen. These are things that could potentially go wrong and what might happen so if it does, you’re not completely blindsided. Here are things you should feel empowered to request.”	“Only thing that was shocking was that I had to get induced, so my water bag had just bust. I didn't know, like the pain was just going to come out the blue like that”

### Perinatal disparities

#### Access to care

Most clinical care team members acknowledged the presence of disparities such as decreased quality of care experienced by Black individuals compared with their NHW counterparts. A clinical care team member stated, “where I feel I see a lot of disparity is not having access to insurance that really decreases the quality of care. There are a lot of restrictions in terms of where patients can go with specific types of insurance or if they don’t have insurance at all.” The interviews also revealed that Black persons are more likely to have fewer appointments and start care later, resulting in delays in tests or missed workups. As one clinical care team member put it, “we see a trend of missed appointments. If they can’t come in their own cars, they won’t come to receive care.”

Patients confirmed this reality, with multiple citing insurance coverage or paperwork as one of their main challenges during pregnancy. While one patient mentioned they had to choose their site of care solely because “that’s where they offered it,” many patients highly valued their ability to choose where and from whom they received care, but some faced barriers to this choice. A patient told a story including a delayed appointment and an unwanted change in provider, relaying “my insurance had canceled because of my age and then she wanted me to pay out of pocket.”

Many patients also found difficulties with accessing and scheduling appointments, one noting, “…and then once you get to doctor's appointments, Oh my God, Scheduling. Because it's like… today is December 7th. Well, [they] have a doctor's appointment for February the second.” Another patient wished providers offered more flexibility in appointment times. Many patients we interviewed travel long distances across Chicago to receive prenatal care, often requiring babysitters for their children or other arrangements. One patient recounted, “I live out South. So I'm like an hour ride to the North side. So when you tell me, Oh, I can't be seen. And I'm going to go all the way that back south to make a new appointment to come all the way back up North, that's just a no, …Especially when I'm on public transportation.”

Long wait-times in- office also contribute to the effort it can take to reach an appointment. One patient says, “it feels like I have to carve out my whole entire day” while another reports sitting in a cold exam room for hours. While this can sometimes be the reality in clinical settings, it is important to note that it is not always easy to fit appointments into a schedule, especially among other inequities that Black birthing people deal with day-to-day. In the setting of prenatal care, issues like these can leave pregnant individuals with long gaps in care at important prenatal stages.

#### Resources

Given these disparities, assistance beyond the current routine prenatal care is often needed to combat challenges like the above that Black birthing people disproportionately face. While a few patients felt they didn’t need additional supports, one shared, “so I was laid off, my job due to the virus… I had to continue to pay every time I had a prenatal visit, I had to pay $40” and that her clinic was able to connect her to external coverage resources. Some patients we interviewed faced challenges in finding childcare, housing, and transportation.

Clinical care team members described the need for addressing SDH, mental health, and contraception and family planning. One clinical care team member mentioned, “Particularly for low-income Black women, having the resources and support to link with social services they need, whether it’s transportation, housing, nutrition support, and mental health challenges and how and when women can access those support services can be a cause for anxiety.” Further examples of resources that benefit Black birthing people are discussed under the category Services and Resources.

#### Racism

Aside from the need for additional physical resources, attention to the experience of Black patients seeking perinatal care is of paramount importance. A theme patients returned to over and over was the importance of providers listening to their Black patients. Some patients emphasized the need for advocates like doulas to combat racial mistreatment, like a patient who acknowledged racism in hospitals and suggested “[having] someone there that can help advocate for you just based off what African-American women have been going through in hospitals.” The racism she refers to may be reflected in the many instances of patients’ concerns being ignored or brushed off reported in the interviews. One patient told about a time when she was “constantly telling everyone the doctors, [everybody], that something’s not right and no one's listening to me.” Another patient noted, “I’ve met some [providers] that just … don't care. They don't listen to you. They just say, this is this and this is your only option.” Still another shared that an anesthesiologist did not take her seriously and comments, “to be ignore[d] for that long, … that's what really troubled me.”

Patients who did report positive prenatal experiences talked about being treated with respect and having their voiced needs heard and taken into account. A patient who had a positive relationship with her provider details, “when I do decline something or I'm apprehensive, she still explains very well and still always lets me know that at the end of the day, it's my choice. A lot of the times with healthcare workers…you just feel powerless sometimes because… they're the doctor and you're not, but I wasn't made to feel that way.” When giving advice to healthcare providers, the interviewed individuals most frequently suggested simply to listen to Black patients. The urgent need for Black birthing people to be listened to is clear with requests like, “If a woman says something is wrong, please just listen to her.”

The longstanding persistence of racism (both historical and current) and discrimination are factors that contribute to these perinatal disparities. As one clinical care team member put it when referencing causes of disparities, “racism and discrimination in the healthcare system… Black women not being listened to in the context of pain or discomfort. When they make those expressions sometimes they’re ignored and other times they are told it’s not that big of an issue. That issue in particular is something that has been happening even in the early days of the study of obstetrics and gynecology—we can sort of track back these institutional practices of healthcare providers where Black women’s pain and discomfort is ignored. It’s unfortunate we continue to see those same patterns today and there have been clear examples where that has actually led to maternal death for Black women.” Several other clinical care team members also mentioned how Black persons are not listened to or treated with respect, and how frequently their pain is ignored. The overall sentiment by clinical care team members was that in order to improve patient care for Black individuals, providers must address trust, respect, making sure patients feel listened to, and cultural connectivity. A clinical care team member with research in eliminating racial disparities in maternal and infant mortality shared, “the cultural humility model focuses on this partnership between patient and doctor as a way to address the racism, bias, and stereotyping and can help eliminate racial disparities in maternal mortality.”

### Trust

#### Importance

Consistent with the quoted statement from the aforementioned care team member, each patient interviewed talked about trust in some way, with most directly emphasizing its importance during their care. Two patients stated that if they did not trust their provider, they would not go to receive care from them. As one patient starkly puts it, “If I didn't trust them, I would not go… if I can't trust you, I know you're going to kill me… it may seem funny, but realistically, yeah.” One patient who did not trust a group of providers shared that “I felt like they treated me like I was a number. They didn't treat me like I actually have a human being in my stomach.”

As notable as the negative impact of lack of trust was the value patients placed on having a provider they *could* trust. One patient recounted a positive experience: “And to know that like I was able to have someone that was able to inform me, educate me, … especially on things that I didn't know, and to answer my questions…. It was definitely uplifting.” Multiple patients said they trusted providers to whom they felt they could ask any question and those who anticipated patient questions and needs.

The importance of trust was also discussed by all clinical care team members (including a majority of them acknowledging the historical context of how Black persons have been treated by the medical community). In fact, one clinical care team member mentioned, “trust is the most core fundamental value that’s missing.” This sentiment was shared by many other clinical care team members. Many described how trust must come before discussing care and that trust can lead to positive behavior change. Specifically, one clinical care team member stated, “trust lays the pathways and foundations for behavior change, and it creates the opportunity for there to be bi-directional change. It creates the opportunity to increase their health literacy and believe that the information that you’re providing is in their best interest.”

#### Establishing trust

For patients, trust seemed to start with building a relationship. Patients felt that interest in their lives, establishment of rapport, and a good tone went a long way in instilling trust. As the relationship progresses, several patients cited honesty, openness, and approaching patient care as a team were crucial to forming a trusting relationship with their providers. Around half the patients interviewed also mentioned some form of follow-up call or check-in as going a long way in building trust.

Most patient interviewees claimed that continuity of care and having an established relationship with a provider contributed strongly to feelings of trust. Several participants had seen their provider or group even before becoming pregnant. Statements like “I will be there with you the whole way” and “she made sure that she was present” when talking about trusted providers emphasizes the importance of longitudinal prenatal care in building trust.

Many clinical care team members discussed how forming trust is an intimate process that involves acknowledging biases with the patient and addressing distrust head on with a trust screening. One clinical care team member described, “if we talked more about trust – do you trust and feel respected by your healthcare provider – having that uncomfortable conversation can help the psychological fear of [physicians not looking out for their best interests]”. This process includes providers learning to understand and not judge patients despite not agreeing with their lifestyle.

A few patient comments emphasized the need for providers to initiate these trust-building conversations. One patient shared that she would have liked her provider to address controversial topics and other “new [information] coming out” about cultural norms and practices during pregnancy that the patient didn’t feel comfortable bringing up herself. Another patient stated that she doesn’t bring up questions unless she has a problem. In contrast, patients who already trusted their providers expressed that they felt they could ask anything. Examples like this emphasize the need for provider proactivity in building trust in order to best care for patients’ needs.

### Change initiatives

#### Education

Patients valued education they received from providers, citing it as one of the reasons they trusted them. One patient noted, “I feel like when a doctor can talk to me, explain things to me…, then yeah. I can trust you when you tell me … next time I see you, [I] want to have this or that.” One of the most frequent comments from those with positive experiences was that the provider answered all off the patient’s questions and the patient felt comfortable asking them anything. On the other hand, some patients had different experiences, reporting instances like, “they didn’t even talk to me. They just gave me medicine and said this what was… wrong.” Some patients valued the brochures and written information while others did not. A few patients noted that they would have liked to be more informed about what the birthing process would be like.

Many clinical care team members felt that patient education is a strength throughout pregnancy and after. Among the most commonly-mentioned change initiatives was teaching patients about important topics including breastfeeding, postpartum contraception, what to expect in postpartum, health literacy, and preeclampsia. In one clinic, a clinical care team member mentioned while discussing important topics covered about breastfeeding “…orient them and prepare them for [breastfeeding] because often people don't understand that, you know, might be really difficult. You might have a hard time, it might take a lot of work. You don't produce enough milk, you're going to feel guilty.” Many clinical care team members also believed focused education of providers about social and health equity could benefit patients.

#### Access to care

In order for education efforts to succeed, patients need to consistently access care. As addressed previously, many Black birthing people face challenges due to disparities in accessing care. Some patients experienced this is the form of disconnection from their practices and providers. In describing a QI project meant to combat this, one clinical care team member stated: “We noticed that moms weren’t coming back for postpartum care. We did a postpartum care pilot project to see what was happening. We used some appointment reminder cards and our case manager at that time to help be the glue between the clinical team and moms. We noticed they used the cards, and I felt like the small reminder did wonders for the return to postpartum care.” Clinical care team members, in general, believed disparities in healthcare needed to be addressed, with foci around ensuring specific clinic staff, services, protocols, and policies that impact Black patients address their needs.

#### Patient advocates

Reiterating an idea brought up by a patient cited in a previous category, many clinical care team members stated the importance of establishing and expanding the use of advocates that patients can trust including, but not limited to, doulas and midwives. A clinical care team member noted, “A lot of these positive examples [initiatives] from my perspective have largely come from community based perinatal support organizations like midwifery care or community-based doulas… they are centering Black women and other women of color in healthcare practice. What I have seen that has been key in their work is that they center women of color and their experiences.”

Two of nine patients did mention doulas as essential sources of support and education during their pregnancies. One patient recounted, “I feel very supported by my doula and how she's set up. Like she even came to my home to do her own prenatal visits with me. It was part of her package. So she does prenatal visits. She of course will support me during and after labor and postpartum. She's gonna still help me with breastfeeding.” Another advised, “I think the best is to have a doula. Get a doula. They help, they help a lot. Cause there's a lot of stuff you're not going to understand.”

### Services and resources

#### Beneficial services

Other services and resources each clinical care team member’s workplace had available for patients varied tremendously. Of note, things that worked and were viewed positively included mental health services, food accessibility services, education services, and holistic care services (midwife led services). In reference to an educational mom group that guides pregnant persons through pregnancy and breastfeeding, one clinical care team member said, “I’ve had patients that have done the moms group and absolutely loved it. They were really invested, motivated, they got a lot of information.” Clinical care team members who did not have these and other services available (including transportation and quick and seamless connection to primary care following birth) cited them as much needed.

Several patients were happy with their clinic’s ability to connect them to needed resources, including ride services, WIC, and prenatal classes. One patient noted that her clinic “provided and offered a virtual, like pregnancy plan where like, I can take pregnancy classes with other people online…” Another found it difficult to get to her appointments across the city and reported that her doctor “went above and beyond and faxed [the company] all my appointments to make it easier for me. To ensure that I had a ride there whenever my appointment was, and I didn't have to call them every time I needed a ride.” Others appreciated connections to nutrition, transportation, and educational services. This highlights the essential nature of integration of these kinds of resources into prenatal care at the sites patients already receive services, as Black birthing people face barriers that can prevent receiving quality care. As an interviewee notes, during pregnancy, “everything will be needed and it’s best to get it when it's… provided for you.”

#### Addressing patient needs via navigation

Patient navigators, who are charged with connecting patients to the above resources, seemed not to be an effective solution to the problem of lower-quality care if they were not fully integrated into their system (face-to-face team interaction and shared EMR access). For these clinical care team members, “patient navigators cannot be a band aid.”

Other clinical care team members described how community services were not helpful or the benefits were unknown at times due to the dearth of services available, the lack of knowledge of providers about what is available, or what the outcome of a patient using a service is. When asked about how beneficial WIC and referrals to primary care physicians were to patients, one resident physician noted, “I can’t say that for any individual patient that I refer to either of these things that I’m able to see the outcome.”

### Social determinants of health

#### Effect on care

Some patients mentioned facing challenges with various SDH during their interviews, sometimes receiving additional support. One patient had “[gone] through being homeless a few times” while others had need for transportation, nutrition, and childcare resources. One patient who was connected with the Women, Infants, and Children (WIC) program during her pregnancy shared, “I was the only aware that it was provided, like after baby was born just for the baby where they, you know, informed me that no, you know, you can get it now. It's also for you too. And once I started getting it, it was a big benefit because, it provided fruits, vegetables, stuff that, that I wanted at that time.”

Clinical care team members also spoke about this program and others focused on SDH. A nutritionist described that Black birthing individuals may “not realize that they can be part of WIC while they’re pregnant.” Our clinical care team interviewees also mentioned that there may be hesitancy to speak up in appointments to avoid being stereotyped, connecting to Black birthing patients’ expressed need to trust their provider. Conversely, some patients when asked what advice they would give other patients responded, “Ask whatever you want,” and directly expressed comfortability asking their provider about anything.

Clinical care team members described the screeners given to their patients and a variety of social factors that disproportionately impact Black health including, but not limited to, lack of access to care, lack of insurance, housing instability/homelessness, and lack of childcare. In reference to differences in SDH, one clinical care team member stated, “particularly for low-income Black women, having the resources and support to link with social services they need, whether it’s transportation, sometimes housing can be a big issue, nutrition support, these are all issues that are related to health.”

#### Best suited to screen

There was no consensus among clinical care team members on the particular role best suited to address these SDH (e.g. social service worker, provider, etc.). While many described that having a social worker is invaluable, responses varied from case workers to all providers to the federal government.

Trust, as discussed above, was the foundation that allowed one patient to share important information about her life with her provider and receive assistance. She said, “I was telling her my situation about me not working and everything with the job. So she recommended me to apply for County care, like a medical card… She gave me the information, I applied online and then I got the card within like two to three weeks.”

Indeed, the likelihood of building and securing trust was a common underlying theme for how to identify the best suited role among clinical care team members. As one resident physician mentioned in reference to who is best suited to address SDH, “I feel like whoever the patient trusts, like whoever [healthcare worker] feels like they’re building rapport with that patient and asking those questions are going to yield meaningful answers.”

#### Frequency of screening

Many clinical care team members stated the need for screenings for SDH to occur throughout pregnancy and at every visit; but almost all of them stated they did not have time to do it themselves. As one physician described adding screenings to her workload, “So if I’m implementing a screening questionnaire, that’s not a waste of my time because the information is important, but I don’t think it’s an optimal use of my time.”

### Perinatal care practices

#### Time devoted to patient care

Attending to these crucial patient needs can often be challenging given constraints in the clinical setting. Some clinical care team members described inefficient use of time when delivering perinatal care with patients or not having enough time with patients. One clinical care team member mentioned how there is too much time spent on “super technical aspects of health” (e.g., genetics) or the EMR and not enough on what is directly impacting their patient (i.e. SDH). Clinical care team members stated a need for extended clinic hours for patients who work late, a need also expressed by one patient, and an appointment scheduled early in the third trimester to discuss labor and what could go wrong. In discussing how to improve perinatal care, one clinical care team member said, “I think we should extend our hours every day instead of just two days a week.” Lastly, a big emphasis was put on postpartum care and its importance.

Patient perceptions of postpartum care varied significantly. Many patients thought of postpartum care as support around primarily birth control and breastfeeding. One patient revealed, “first thing they talk about is the birth control.” Patients also mentioned postpartum depression and C-section follow-up. Support took the form of follow-up calls, brochures, and appointments. Most who had received postpartum care were satisfied with it, but on patient remembered pressure from her provider around breastfeeding: “I just felt like I had already made up my decision that I wasn't going to do it, but then she just kept pushing it and pushing it and pushing and pushing it.”

Several patients also mentioned the importance of postpartum SDH supports like WIC, childcare, and mental/emotional support. A patient noted, “But most importantly, … they gave you the things in the tools you needed to know,” emphasizing how critical postpartum care is. Two of nine patients, however, reported receiving no postpartum care or advice, and several responded to the term “postpartum” only referring to postpartum depression, indicating a need for more comprehensive postpartum education and care.

#### Cultural responsiveness

Part of comprehensive care includes leveraging the experiences and ideas of patients themselves to inform culturally responsive care. The majority of patients believed strongly in their own intuition and accountability in maintaining a healthy pregnancy. Many talked about drawing information from their own previous experiences, bodily feelings, research, and familial knowledge. One patient said, “So I know by now what should be going on with my body when I'm pregnant.” The value of patient experience and knowledge must be taken into account to provide culturally responsive care.

Clinical care team members dedicated most of this section to describing patient education needs and cultural competency. One clinical care team member mentioned, “You have providers who are not accustomed to working with African American patients who don’t always understand dialects/lingo and don’t utilize language that patients can always understand, which can have a major impact on patient education and their understanding of what exactly their medical condition is.” The need to focus on educating younger, new moms was described, while also realizing that multiparous persons should not be expected to know everything about pregnancy.

Patient opinions reflected the need for education and responsiveness to both populations. One patient thought it important to support first-time pregnant persons by “…be[ing] aware of the new stuff and …each generation’s change.” A patient who had other pregnancies expressed the need for ongoing support and education: “even when you think you’re a veteran mom, you think you know, but you don't cause everything changes over the years.”

#### Mental health

Most patients focused on the physical aspects of prenatal care, with many citing the progress of the pregnancy and health of the baby as the most important part of prenatal care. A few patients, however, did talk about the importance of attention to mental and emotional health in the perinatal period. When asked about what prenatal care means to her, one patient responded, “prenatal care is seeing to it that I am all around taken care of physically and emotionally.”

Clinical care team members reported that referrals were often available for patients to learn about and address mental health needs, but there is sometimes stigma around Black persons’ mental health. As one clinical care team member put it, “Some of the cultural stigma around mental health and strong black woman syndrome… can really be a barrier in terms of acknowledging that there are challenges [with mental health] and that we should talk to someone.”

#### Expectations for labor and delivery education

A few patients mentioned that they would have liked to be more informed about the birthing process before actually going into labor. One said, “Only thing that was shocking was that I had to get induced, so my water bag had just bust. I didn't know, like the pain was just going to come out the blue like that,” and that she would have liked “more information” previously. One patient received a booklet from her provider about the process but would have liked more information. Some patients liked the printed resources while others did not use them. Many found birthing classes or doulas helpful in making expectations for labor and delivery clearer.

For many providers, it was of utmost importance to have more time to discuss the course of the pregnancy and the normal body changes that will occur. “I think a lot of expectations in labor and postpartum we should address ahead of time, globally for patients, but especially for patients that might not have as much trust in the health system… maybe around 30, 32 weeks, labor wise, here’s all the things that might happen. These are things that could potentially go wrong and what might happen so if it does, you’re not completely blindsided. Here are things you should feel empowered to request.”

As the pregnancy progresses, more time to discuss expectations in labor and postpartum could help pregnant patients and their partners prepare for potential hardships, as the above clinical care team member described. One of the key topics to discuss during this time is postpartum contraception to ensure patients know all of their options. A clinical care team member stated, “they [postpartum women] lack the support that they need trying to secure access to contraception or family planning services.” Most patients did report receiving counseling or provision of postpartum contraception, but several did not.

## Discussion

An understanding of both patient and other stakeholder perspectives on perinatal care disparities that Black persons endure is critical for the development of sustainable interventions, practices, and policies that address the real-life experiences of these patients. With samples of both patients and multidisciplinary care team members, this study aimed to explore and document perceptions of care that Black pregnant individuals receive from different viewpoints and use that information to guide the development of an intervention intended to improve Black pregnant individual’s receipt of recommended perinatal care components. Overall, patients emphasized multiple barriers to care and the importance of listening and trust in the patient-provider relationship. Clinical care team members described a longstanding discrimination of Black persons in healthcare and the distrust that stems from that history. Our study findings add nuance and rich detail as explained by patients and clinical care team members to the current literature on perinatal care experiences of Black persons in the U.S. and give direction on how to improve care.

The findings have important implications and provide insights on how to approach the development of our OPTIMIZE perinatal care improvement intervention that aims to enhance the health of Black persons during pregnancy. Alongside patient navigators – who make appointment reminder calls; provide interpreter services; refer patients to appropriate community services; and provide logistical and emotional support – there needs to be trust in provider-patient relationships in order to maximize the benefit of healthcare experiences. According to clinical care team member feedback, an allotted time to get to know patients and their goals for the pregnancy needs to be established in the first prenatal visit. Questions like “what are your goals for this pregnancy,” “what are your hopes about how this pregnancy will be,” and “what are your concerns for this pregnancy,” should be asked. Our Advisory Committee’s interpretation of the results felt this included asking identity questions including pronoun, gender, and sexual orientation. In addition, a review of SDH should be completed to learn more about patients’ unique needs. In each subsequent visit there should be check-in questions that include “how are you doing now,” “did you have any questions from the last visit that you didn’t get to ask,” and “do you have any concerns about your pregnancy or care experience that you’d like to discuss today with me?” The belief is that these interventions not only can help build trust, but also ultimately improve perinatal outcomes in Black persons. The OPTIMIZE study will help evaluate if these trust building exercises provide improvement in care.

The results convey that the current approach to addressing SDH varied greatly between providers. There was a general consensus in feedback suggesting that those who the patient trusts most should be administering screening. Patients expressing the paramount importance of trust solidifies the need for careful attention to these relationships. These results have important implications for the intervention above. The expected increase in trust begets more disclosure on social circumstances that could be difficult for patients. Thus, the provider who is having the trust conversations with the patient should also ask about SDH.

Our results describe patients’ and clinical care team members’ personal experiences related to what is now well known – Black persons experience worse perinatal health outcomes including a three- to four- times greater likelihood to die from pregnancy-related causes [35]. In our study, real patient experiences and reasons believed by clinical care team members to be driving the differences in perinatal care included poor insurance coverage or no insurance that may affect ability to appropriately follow up, appointment accessibility issues, the longstanding persistence of racism and discrimination towards Black individuals, and concerns of being ignored. These reasons for worse care are consistent with studies showing that poor communication and insurance type can lead to suboptimal care and often stem from discriminatory practices [36]. It is important to note that these patient and clinical care team member perspectives do not necessarily represent all experiences of Black birthing people.

Our results align with research on the evidence base for interventions to reduce health disparities, and on the role of trust in particular. Patients expressed that they needed providers they trust and clinical care team members described how trust can lead to higher engagement in a patient’s own care and a belief that the care being provided is truly for the patient’s benefit. These results are similar to other studies that have found some level of distrust in healthcare providers that may lead to unwillingness to follow health recommendations [36]. The history of discrimination and racism towards Black persons in healthcare was also described in our interviews for reasons leading to distrust. To help establish trust in this patient population, clinical care team members believe that being proactive and facing bias head on can help. Patients believe honesty, continuity in the relationship, and a good demeanor go a long way in trust-building, and that providers should often be the ones initiating this process. Building rapport early on by learning about a patient, making a judgment free space for questions to be asked, including what goals they have for the pregnancy, can help foster a good relationship between provider and patient. Prior research corroborates many of our findings related to trust. Actions that were shown to build trust early included asking patients about their goals, avoiding language and behavior that is judgmental of patients, and emphasizing that it’s okay for patients to ask questions [37]. Patients’ expressed need to ask questions and have them answered frequently supports this conclusion.

Additionally, our study suggests there are a variety of change initiatives happening within health centers. The diverse set of initiatives that had a similar goal of reducing Black persons disparities in healthcare spanned from policy change to education to expanding patient support before, during, and after pregnancy. Literature supports that quality initiatives – like the ones mentioned by clinical care team members – aimed at standardizing delivery care is likely to improve care for racial minoritized persons [35]. Additionally, many patients and providers alike found it valuable to include an advocate for the patient such as doulas or midwives. Studies have corroborated the impact of doulas on healthy birth outcomes including significant reductions in cesarean births, instrumental vaginal births, need for oxytocin augmentation, and shortened durations of labor [38]. Furthermore, doula support has been associated with higher newborn Apgar scores and greater satisfaction with the birthing process compared with those without doulas [38]. In addition, mental health services, food accessibility services and education services were valuable to some patient interviewees and thought to be highly useful for patients by clinical care team members. When considering what is not working well within some clinic systems, a common sentiment was services that are not well integrated were not as successful (e.g. community services that needed referrals out).

Key limitations should be noted. First, the data represent a purposive sample of patients and other care team members recruited from community partners. Our sample size was relatively small. Our sample included only individuals who identified as Female or did not answer. This excludes many valuable perspectives from other genders. We recommend caution in generalizing this study in other settings. Second, the interviews assessed past interactions that may be affected by social desirability bias, recall bias, or inaccuracies. This study solely focused on the experiences of some patients and clinical care team members, but these may not match the true perinatal experiences of Black persons. The study evaluated these perspectives of perinatal care disparities to determine areas that are critical to developing a sustainable intervention for the real-life experiences of Black persons from a provider perspective. Our team acknowledges that some of the comments made by providers overgeneralize in ways that are rooted in systemic and structural racism and implicit bias. Despite these limitations, this study is playing an important role in both guiding development of perinatal care interventions and adding nuance to the understanding of Black pregnant and birthing person’s healthcare experiences.

## Conclusion

In summary, this qualitative study exploring patients’ and clinical care team members’ experiences caring for Black pregnant and birthing persons identified potential approaches to eliminate health inequities, most importantly in the form of building trust and forming relationships. These findings have direct implications for developing perinatal care interventions in which trust building questions will be incorporated into clinic visits.

## Data Availability

The datasets used and/or analysed during the current study are available from the corresponding author on reasonable request.
